# From Loss to Healing: Navigating Perinatal Grief with Enhanced Psychological Care

**DOI:** 10.1192/j.eurpsy.2024.490

**Published:** 2024-08-27

**Authors:** K. Razki, Y. Zgueb, S. Ben Aissa, C. Najar, U. Ouali

**Affiliations:** psychiatry department, Razi hospital, manouba, Tunisia

## Abstract

**Introduction:**

Several factors can influence the journey of perinatal grief in mothers, including the quality of care during this experience. The objective of our study was to investigate the factors influencing the perception of grieving women during the perinatal period and identify the role of medical and paramedical healthcare professionals in psychological support.

**Objectives:**

To determine the factors influencing the perception of fetal loss in grieving mothers.

**Methods:**

This was a descriptive, longitudinal, retrospective study conducted between july 2021 and march 2022 at the Fetal Pathology Department of the Center for Maternity and Neonatology in Tunis, Tunisia. The study included women who experienced perinatal loss and underwent fetal pathology examination.

The study was conducted in two stages: Initial consultation at the Fetal Pathology Department, five weeks after the date of expulsion, for perinatal grief counseling. Follow-up interview one year after the date of expulsion: The participants were contacted via telephone for an average duration of twenty minutes .The assessment of perinatal grief during both interviews was conducted using the shortened version of the Perinatal Grief Scale (PGS)

**Results:**

The mean age of the patients was 31.41 years (± 5.15). The average gravidity was 2.47 (± 1.43). More than half of the patients had no living children (n=41). The majority of patients had no notable pathological history. Six patients had been followed in psychiatry, and five had a history of subfertility. The majority of patients (n=61) reported having good marital relationships.

Among the participants, 20% (n=14) had a high Perinatal Grief Scale (PGS) score (PGS >= 91) at five weeks post-loss and were subsequently referred for psychiatric consultation.

At one year, all participants had a PGS score > 91, demonstrating the effectiveness of psychiatric management. Multivariate analysis identified four independent factors associated with a high PGS score at five weeks: absence of living children (OR=0.59; 95% CI [0.36-0.98]; p=0.04), quality of marital relationship (OR=1.2; 95% CI [1.1-3.9]; p=0.02), family support (OR=2.52; 95% CI [1.55-4.12]; p<0.001), and quality of loss disclosure (OR=2.52; 95% CI [1.32-3.77]; p=0.003).

**Conclusions:**

To identify patients at high risk of developing complicated grief and improve the quality of psychological care, it is necessary to implement appropriate protocols, provide training to healthcare personnel, and establish well-equipped healthcare facilities.

**Disclosure of Interest:**

None Declared

